# The Relationship between Cocaine Use and Human Papillomavirus Infections in HIV-Seropositive and HIV-Seronegative Women

**DOI:** 10.1155/2008/587082

**Published:** 2008-04-15

**Authors:** Howard Minkoff, Ye Zhong, Howard D. Strickler, D. Heather Watts, Joel M. Palefsky, Alexandra M. Levine, Gypsyamber D'Souza, Andrea A. Howard, Michael Plankey, L. Stewart Massad, Robert Burk

**Affiliations:** ^1^Department of Obstetrics and Gynecology at Maimonides Medical Center and SUNY Downstate, NY 11219, USA; ^2^Department of Epidemiology and Population Health, Albert Einstein College of Medicine, Yeshiva University, Bronx, NY 10461,USA; ^3^Pediatric, Adolescent and Maternal AIDS Branch of National Institute of Child Health and Human Development (NICHD), National Institutes of Health, Bethesda Maryland, MO 20892, USA; ^4^Department of Medicine, The University of California, San Francisco (UCSF), CA 94143, USA; ^5^Department of Internal Medicine, University of Southern California School of Medicine, Los Angeles, CA 90033, USA; ^6^Department of Epidemiology, Johns Hopkins Bloomberg School of Public Health, Johns Hopkins University, Baltimore, MD 21205, USA; ^7^International Mailman School of Public Health Columbia University, NY 10032, USA; ^8^Department of Medicine, School of Medicine, Georgetown University, Washington, DC 20007, USA; ^9^Department of Obstetrics and Gynecology, Southern Illinois University School of Medicine, Springfield, IL 62794, USA; ^10^Department of Pediatrics, Albert Einstein College of Medicine, Yeshiva University 1400 Pelham Parkway South Bronx, NY 10033, USA

## Abstract

*Objective*. Animal data suggest that cocaine has an immunosuppressive effect, but no human studies have been conducted to assess the relation of cocaine use with human papillomavirus (HPV) infection, the viral cause of cervical cancer. Since both cocaine use and HPV infection are common among HIV-positive women, we sought to determine whether use of cocaine and/or crack influences the natural history of HPV among women with or at high risk of HIV. *Methods*. Women enrolled in the Women's Interagency HIV Study (2278 HIV-seropositive and 826 high-risk seronegative women) were examined every six months for up to 9.5 years with Pap smear, collection of cervicovaginal lavage (CVL) samples, and detailed questionnaires regarding health and behavior, including use of crack and cocaine (crack/cocaine). CVLs were tested for HPV DNA by PCR, with genotyping for over forty HPV types. *Results*. In multivariate logistic regression models, censoring women treated for cervical neoplasia, crack/cocaine use within the last six months was associated with prevalent detection of oncogenic HPV DNA (odds ratio [OR] = 1.30 (1.09–1.55)), and with oncogenic HPV-positive squamous intraepithelial lesions (SIL) (OR = 1.70 (1.27–2.27)), following adjustment for age, race, HIV-serostatus, and CD4+ T-cell count, the number of sexual partners in the past six months, and smoking. In multivariate Cox models crack/cocaine use was also associated with a trend that approached significance in regard to incident detection of oncogenic HPV-positive SIL (HR = 1.51, 95% CI 0.99–2.30), and while the rate of oncogenic HPV clearance was not related to cocaine use, the clearance of any SIL was significantly lower in those with versus those without recent crack/cocaine use (HR = 0.57, 95% CI 0.34–0.97). *Conclusions*. Cocaine use is associated with an increased risk of detection of both prevalent and incident oncogenic HPV infection, as well as an increased risk of HPV-positive SIL over time.

## 1. INTRODUCTION

Human papillomavirus (HPV) infections of women, though common, typically resolve. Persistence of oncogenic HPV is necessary for the development of cervical precancer and invasive cervical cancer (ICC). The incident detection and persistence of HPV infections and related lesions have been shown by us [1–4], and others [5–8], to be elevated among HIV-seropositive compared to HIV-seronegative women, and to be strongly associated with biomarkers of immune status such as CD4+ T-cell count and HIV RNA level in HIV-infected women.

Cocaine use is also common among HIV-infected women [9, 10] and has been linked to perturbations of the immune system in both animal models and humans [11–13]. In one study of lymphocytes from individuals with cocaine in their urine, it was found that “Memory” CD8+ T cell subpopulations (i.e., CD45RO+) were reduced in the cocaine-positive patients leading the authors to conclude that this might “represent a disruption of particular immunologic cell networks which could ultimately influence host resistance to infection” [14]. Other researchers have suggested that cocaine could inhibit the effector functions of neutrophils and macrophages, thus interfering with the host's ability to defend against infections, as well as suppressing cytokine production, thereby diminishing the capabilities of additional immune cells important in control of viral infection [15–17].

Any disruption to host defenses attributable to cocaine, in conjunction with those associated with HIV-infection per se, could increase the incidence and/or duration of oncogenic HPV infection. That in turn could contribute to an enhanced risk of cervical neoplasia to which HIV-infected women are demonstrably prone [18]. Additionally, if cocaine use was found to modify the course of an infectious disease (such as HPV), it would provide clinical evidence in support of the assertion of researchers who, on the basis of results in animal models, have expressed concerns about a potentially enhanced susceptibility to infections among cocaine users in general. In this paper, we take advantage of a large, well-characterized cohort of HIV-infected and at risk women to assess the association of cocaine use with HPV infection and disease.

## 2. MATERIALS AND METHODS

The Women's Interagency HIV Study (WIHS) is a prospective study of the natural history of HIV infection in women that initially enrolled HIV-seropositive and at risk seronegative women participants at six clinical sites (Bronx, NY; Brooklyn, NY; Chicago; Los Angeles; San Francisco; Washington, D.C.) between October 1994 and November 1995. A subsequent recruiting cycle occurred in 2002. The recruitment methods and data collection procedures for WIHS have previously been described [19]. In brief, participants underwent visits every six months that included an interviewer-administered questionnaire, a physical examination, and the collection of specimens, including cervicovaginal lavage for HPV DNA testing. At each visit participants were asked about drug use (types and frequency of use). At baseline, they were asked about lifetime use as well as use in the last six months. Questions were asked specifically about cocaine and crack, as well as heroin and alcohol use. Those who acknowledged the use of the drug were asked about the average frequency of use (e.g., once a month, once a week, once a day, etc.) over the last six months.

There were a total of 3768 subjects in WIHS. We excluded 16 subjects who had HIV seroconverted during follow-up, and 253 subjects who did not have a cervix present at baseline. Thus 3499 women were included. We censored all the visits after hysterectomy. The current analysis includes women recruited in 1994/1995 and additional women recruited in 2002. The initial recruits were tested for HPV infection during visit 1 through visit 15, and the more recent recruits were tested for HPV infection during their baseline visit (V15 or V16) through visit 19. Eighty-nine (9.7%) of the 915 HIV-seronegative women and 306 (11.8%) of the 2584 seropositive women did not have HPV DNA test results available and were excluded from analysis. Comparisons were made between HPV carriage patterns (defined below) of women who did or did not acknowledge cocaine use.

### 2.1. Laboratory methods

The laboratory technique for HPV assessments have been described in detail elsewhere [20]. HPV DNA was detected utilizing L1 consensus primer MY09/MY11/HMB01 polymerase chain reaction (PCR) assays [20–22]. Control primer set PC04/GH20, amplifying a 268 bp cellular beta globin DNA fragment, was included in each assay to serve as an internal control for amplification. Following proteinase K digestion, 2–10 *μ*L of each cell digest was used in reactions containing 10 mM Tris-HCL, 50 mM KCL, 4 mM MgCl_2_, 200 *μ*M of each deoxyribonucleotide triphosphate, 2.5 U Taq DNA polymerase, 0.5 *μ*M of each primer. There were 40 amplification cycles (95°C for 20 seconds, 55°C for 30 seconds, and 72°C for 30 seconds, with a 5-minute extension period at 72°C on the last cycle). Amplified material was then detected using filters individually hybridized with biotinylated type-specific oligonucleotide probes for multiple HPV types including HPV 6, 11, 16, 18, 26, 31, 32, 33, 35, 39, 40, 45, 51, 52, 53, 54 (AE9), 55, 56, 58, 59, 61, 62, 66, 67, 68, 69, 70, 71 (AE8), 72, 73 (PAP238A), 81 (AE7), 83(PAP291), 82v(AE2), 84 (PAP155), 85 (AE5), and 89 (AE6), as described in [20, 23]. For these analyses, oncogenic and nononcogenic HPV types were defined in accordance with recently published data that classified types utilizing both phylogenetic and epidemiologic criterion. The oncogenic HPVs were HPV-16, 18, 31, 33, 35, 39, 45, 51, 52, 56, 58, 59, and 66, and all other HPV types were considered nononcogenic HPV [24].

Pap smears were collected using a plastic Ayre spatula and an endocervical brush. Both specimens were smeared onto a single slide and fixed immediately. Cervical cytology samples from all sites were centrally interpreted at Kyto Meridien Laboratories (New York , NY) using the 1991 Bethesda System criteria for cytologic diagnosis [25]. All smears were read by two cytotechnologists who were blinded to the participants' status. Ten percent of all negative smears and all abnormal smears were confirmed by a cytopathologist. Diagnoses were reported as normal or benign, atypical squamous cells of uncertain significance (ASCUS), low-grade squamous intraepithelial lesion (LGSIL), moderate- or high-grade squamous intraepithelial lesion (HGSIL), and cancer. Glandular abnormalities were not included. Whenever possible the primary cytologic outcome used was SIL among women with oncogenic HPV. This was chosen since SIL in the presence of HPV is more clearly a precursor of cancer than are cases of SIL in which oncogenic HPV is not detected. In cases in which the number of such outcomes was sparse, we did use SIL among all women as an outcome.

T-cell subsets were determined by immunofluorescence using flow cytometry in laboratories participating in the AIDS Clinical Trials Quality Assurance Program. Plasma HIV RNA levels were measured using a nucleic acid sequence-based amplification (NASBA) technique (Organon Teknika, Durham, NC, USA) with a lower threshold of detection of 4000 copies/ml [26].

### 2.2. Statistical analysis

Cocaine used in the last six months was examined both as a binary outcome (use/no use), and as an ordinal variable (no use/less than once a month/once a month—once a week/2–6 times a week/once a day or more). Consistent with prior studies [4] the prevalence of HPV DNA was defined as the fraction of women with detectable HPV among those with adequate HPV test results (as determined by the successful amplification of the human *β*-globin gene—see Laboratory Methods). We defined “incident detection” of HPV as detection of an HPV type that was not found at baseline or at any other earlier visit in a given woman. The term “incident detection” is used instead of “incidence” to reflect the fact that it is impossible to distinguish newly acquired HPV infection and reactivation of previously acquired latent HPV infection in a population with many years of sexual activity, regardless of the HIV status of that population. Persistence of HPV infection was defined as the time to clearance of a specific HPV type, and we further distinguished in our analysis between the persistence of HPV detected prevalently at baseline and those first detected incidentally at a subsequent visit (see below). Clearance itself was defined as either a single negative or two negative type-specific HPV results at sequential visits. The findings were similar using either approach for defining clearance, and herein we present the results of clearance using the former definition.

The baseline characteristics of cocaine users and nonusers in this study were examined, and the distributions for selected parameters compared using standard Pearson chi-square tests for categorical data, or by the Cochran-Mantel-Haenszel tests when examining trends in ordinal data. These variables were also evaluated as potential confounders in the multivariable analyses described below.

The association of cocaine use with prevalent HPV DNA detection was measured in multivariate logistic regression models that incorporated generalized estimating equations (GEE) to adjust the standard errors for repeated measures (i.e., summarizing data across all HPV types and across study visits), and assuming an independent correlation structure. In previous papers, we have shown that the results in this dataset are unaltered by using other possible correlation structures [4, 27], and, in general, model estimates are robust with respect to the correlation structure imposed [28].

Treatment of abnormal cervical cytology can introduce a bias into analysis of HPV infection, since treatment can alter the underlying mucosa; either increasing infection (e.g., by exposing basal cells which are the cells susceptible to infection) or decreasing infection (e.g., by removing infected cells or inducing the replacement of columnar with squamous tissue in the transformation zone of the cervix). Censoring women at the time of cervical treatment is commonly employed to address this bias and was used in the analyses reported herein. Adjusting for therapy is an alternate approach that was also used, but as the results were in the same direction and of similar magnitude to those found with censoring they are not presented. Other time-dependent covariates included in our multivariate models were CD4+ T-cell count, recent sexual behavior, smoking status, and age group. Ethnicity was adjusted as a time-independent variable.

Cox proportional-hazard regression models [29] were used to assess the association of cocaine use and the incident detection of HPV. The event time was defined as the midpoint calendar date between the last HPV-negative and the first HPV-positive visit. Since each woman had been tested for multiple HPV types at each visit time, Wei Lin Weissfeld method was applied to adjust for possible correlations between multiple incident infections [30]. A similar method was used to measure the association of cocaine use with time to clearance of HPV, stratified by whether the HPV type was prevalent at baseline or was first detected during follow-up (incident detection). Time-dependent and time-independent covariates, as well as methods for controlling for treatment of cervical neoplasia, were as described above.

Prevalent SIL and its association with cocaine use was assessed using similar multivariate logistic regression models incorporating GEE, as described above, to control repeated observations over time. The associations of cocaine use and incident detection of SILs were assessed using multivariate Cox models.

## 3. RESULTS

This analysis included data from 2584 HIV-seropositive and 915 HIV-seronegative women. The median follow-up time among those recruited during 1994-1995 was 10 years and for those recruited during 2002 was 3 years; a total of 20 849 person years of observation among HIV-seropositive and 6385 among HIV-seronegative women were included in this analysis. At baseline 16.1% of participants reported use of crack within the prior 6 months, 11.5% had used cocaine, and 6.9% had used both. The prevalence of HPV in the cohort was 34.4%. Although we initially analyzed the effects of cocaine use on HPV infection separately from the effects of crack use, no significant (or otherwise notable) differences were observed (data not shown). Therefore, we combined these data, and herein report a comparison of the prevalence, incidence and time to clearance of HPV among women who used either cocaine or crack (crack/cocaine use) to that among women who used neither drug.

Table 1 shows the demographic characteristics of the study population at baseline. Women acknowledging crack/cocaine use were older, more likely to be African Americans, less likely to be Hispanic, had more sex partners in the last six months, and were more likely to report heavy cigarette use. Among women who reported crack/cocaine during the past 6 months, 18% reported using less than once per month (<once/mo), 31% reported using between once/month and once/week (once/mo-once/wk), 25% 2–6 times per week (2–6/wk), and 25% once per day or more (≥once/day).

Prevalent detection of oncogenic HPV was significantly more common among women who reported crack/cocaine use during the prior 6 months (Table 2), than women who did not use either drug (OR = 1.30; [95% CI: 1.09–1.55]), after adjustment for age, race, HIV serostatus and CD4+ T-cell count, the number of sexual partners during the prior 6 months, and smoking behavior, all factors that we found to be associated with cervical HPV detection in prior studies [4, 31]. Nononcogenic HPV was also more prevalent among crack/cocaine users; OR= 1.18 (1.04–1.34). Oncogenic HPV-positive cervical neoplasia (i.e., squamous intraepithelial lesions [SIL] at risk of progression [oncogenic HPV+ SIL]) was also significantly more common among women who reported crack/cocaine use during the prior 6 months (Table 2; OR = 1.63; 95% CI 1.20–2.21).

Incident detection of oncogenic HPV was increased among crack/cocaine users, although the relationship did not reach significance at traditional values (Table 2; hazard ratio [HR] = 1.21, 0.97–1.52). Similar results were observed for incident detection of nononcogenic HPV and any HPV (Table 2). Crack/cocaine was also associated with a trend that approached significance in regard to an elevated risk of incident oncogenic HPV+ SIL (OR 1.51; 95% CI: 0.99–2.30).

Clearance of oncogenic HPV infection was not associated with crack/cocaine use during the prior 6 months (Table 2). Similarly, no association was found between cocaine use and clearance of HPV+ SIL, although the data were sparse given the more limited occurrence of this endpoint (data not shown). Therefore, we assessed associations between cocaine use and any SIL (with or without oncogenic HPV). We found a significant association between delayed clearance of SIL and cocaine use, (HR 0.57; 95% CI 0.34–0.97). No statistically significant dose response was seen with any of the outcomes when frequency of cocaine use was assessed as an ordinal variable (data not shown).

Similar analyses were performed for alcohol and heroin use. No association was found between either drugs (whether we considered exposure as a dichotomous or as an ordinal variable) and any of the outcomes of interest (oncogenic or nononcogenic HPV, or SIL).

## 4. DISCUSSION

In this prospective study of HIV-infected and HIV-uninfected women, we observed increased risk of oncogenic and nononcogenic HPV infection in those with crack/cocaine use but not in those with heroin or alcohol use, consistent with our a priori hypothesis. If correct, the data suggest that the immunologic effects of cocaine, previously reported primarily in animal models, may have relevance to humans.

Shen and colleagues, evaluating a mouse model in which cocaine was injected intraperitoneally found that all immune parameters, other than lymphocyte transformation of the splenic or the peripheral blood lymphocytes, were suppressed [11]. Other investigators have shown that withdrawal from cocaine can also induce deleterious immune alterations. Using a rat model, Avila demonstrated that repeated exposure to cocaine followed by withdrawal led to activation of the neuroendocrine stress response, which alters cellular immunity and possibly contributes to an increased susceptibility to infection [13]. HPV in humans is felt to be controlled, at least in part, by an intact mucosal immune system [32]. Perhaps most germane to our findings is the work of Lopez [33] and colleagues, who found that daily cocaine administration induced a significant decrease in the number of IgA+ cells with a concomitant increase in the number of CD8+ cells per villi in the intestinal lamina propria (ILP). Murine retrovirus infection alone decreased the number of IgA+ and CD4+ cells in the ILP, and this decrease was even more marked when murine acquired immune deficiency syndrome (MAIDS) mice also received cocaine. The authors concluded that, “cocaine administration could potentiate the dramatic effect that MAIDS infection has on the mucosal-associated lymphoid tissues” [33].

These data suggest that response to HPV infection might be altered by cocaine. While HIV-infected women are already at immunologic jeopardy and prone to numerous coinfections, the relationship between cocaine use and HPV, one of the most common sexually transmitted infections, has not, to our knowledge, been the focus of prior research. While we and others have considered drug use as a covariate in studies of HPV infections, those studies have often not looked specifically at cocaine or crack, controlled for other factors, or used drug use as a primary focus of analysis [20]. Our data appear to confirm the immunomodulatory effects of cocaine, and suggest that further clinical/laboratory research is indicated to better understand the associations between cocaine use and the detection, and natural history of HPV infection as well as the mechanism that underpins the relationship.

This study had several limitations. The finding of increased detection of HPV among women acknowledging cocaine or crack use could reflect either an immunologic phenomenon or a behavioral one (i.e., increased sexual activity) despite our efforts to control this in our analyses. However, we did not find a similar relationship between HPV and the use of other drugs that might also be markers of risk taking. Our failure to detect a consistent biologic gradient in cocaine's effect (there was an increasing effect with increasing exposure except at the highest strata when sample size became small; data not shown) suggests that caution must be exercised in interpretation of results and that further work is warranted to confirm and potentially extend these findings.

An additional limitation to this study is our inability to provide biologic measures of drug exposure. We note, however, that the same study personnel performed repeated interviews over the course of the study and formed a close rapport with the WIHS participants. Further, we have previously observed a close correlation of acknowledged drug use and reliable surrogates (e.g., use of injection drugs and hepatitis C infection, data not shown). More importantly, it is most likely that misclassification would have been in the direction of understating the frequency of drug use, which would most likely have biased our results toward the null. A final concern is that, since we have previously shown that HAART use can modify the course of HPV disease [34], it is possible that some of the perceived effect of cocaine may have been mediated by a lower rate of medication adherence among cocaine users. To investigate this possibility, we repeated the analyses described above, but limited only to the time frame prior to the availability of HAART. All findings were in the same direction (data not shown) as reported for the time frame after HAART became available. Thus, compliance with HAART use would not seem to be an explanation for our findings.

In summary, our data suggest an association of crack/cocaine use with increased prevalence, incidence, and delayed time to clearance of oncogenic HPV among HIV-infected women and women at risk of HIV. These data lend support to animal studies suggesting a link between cocaine use and predilection to infections and add to the litany of reasons for making drug treatment a component of the care of HIV-infected individuals.

## Figures and Tables

**Table 1:** Selected baseline characteristics of cocaine users and noncocaine users in last 6 months at baseline in the Women's Interagency HIV Study*.

Characteristic	N (%)		*P*-value
	Used crack/cocaine^1^ in last 6 months		
	Yes	No	
Total	726 (%)	2761 (%)	—

Age (yr)			<.0001**
<30	116 (16)	914 (33)	—
30–34	160 (22)	680 (25)	—
35–39	207 (29)	607 (22)	—
40–44	171 (24)	339 (12)	—
>=45	72 (10)	221 (8)	—

Race			<.0001
White	95 (13)	433 (16)	—
Hispanic	121 (17)	829 (30)	—
African American	494 (68)	1397 (51)	—
Others	16 (2)	102 (4)	—

HIV status and CD4 T-cell count			0.0559**
HIV-negative	218 (31)	694 (26)	
HIV-positive			
CD4>500	150 (21)	664 (25)	—
200≤CD4≤500	212 (30)	859 (32)	—
CD4<200	121 (17)	490 (18)	—

Number of male sex partners in past 6 months			<.0001**
0	155 (22)	772 (28)	—
1 (married)	80 (11)	506 (18)	—
1 (single)	223 (31)	1049 (38)	—
2	97 (14)	266 (10)	—
>2	160 (22)	159 (6)	—

Smoking status			<.0001**
None	60 (8)	1106 (40)	—
Former smoker	37 (5)	483 (18)	—
Current smoker <10 pack-years	282 (39)	706 (26)	—
Current smoker >=10 pack-years	344 (48)	456 (17)	—

Frequency of crack/cocaine use			
Less than once per month	131 (18)	NA	—
Once per month—once per week	224 (31)	NA	—
2–6 times per week	185 (25)	NA	—
Once per day or more	180 (25)	NA	—

*Limited to those contributing data to the present analysis. Some data were missing at baseline for selected patients. Percentages do not always add up to 100% due to rounding. **Two-sided Cochran-Mantel-Haenszel test. All other *P* values were determined with the two-sided Pearson's chi-square test.

**Table 2:** Association of crack and cocaine use with prevalence, incidence, and clearance of human papillomavirus (HPV) and cervical squamous intraepethelial lesions (SIL) (adjusted analysis*).

Crack or cocaine use in the past six months	Any HPV	Oncogenic HPV	Nononcogenic HPV	SIL with oncogenic HPV
Prevalent HPV and SIL: OR (95% CI)				
No	1.0	1.0	1.0	1.0
Yes	1.22 (1.09–1.37)	1.30 (1.09–1.55)	1.18 (1.04–1.34)	1.70 (1.27–2.27)

Incident HPV and SIL: HR (95% CI)				
No	1.0	1.0	1.0	1.0
Yes	1.20 (1.02–1.42)	1.21 (0.97–1.52)	1.20 (1.00–1.44)	1.51 (0.99–2.30)

Clearance of HPV and SIL**: HR (95% CI)				
No	1.0	1.0	1.0	1.0
Yes	1.02 (0.88–1.17)	0.96 (0.80–1.16)	1.05 (0.88–1.25)	0.57 (0.34–0.97)

*Adjusted for CD4 count, number of sexual partners, smoking, age and race. **As there were a limited number of SIL infections with oncogenic HPV for analysis of SIL clearance, results presented for risk of SIL clearance include all SIL observed with or without oncogenic HPV.
